# ECG Identification For Personal Authentication Using LSTM-Based Deep Recurrent Neural Networks

**DOI:** 10.3390/s20113069

**Published:** 2020-05-29

**Authors:** Beom-Hun Kim, Jae-Young Pyun

**Affiliations:** Department of Information and Communication Engineering, Chosun University, Gwangju 61452, Korea; godseng1210@gmail.com

**Keywords:** RNN, ECG, biometrics, identification, signal processing

## Abstract

Securing personal authentication is an important study in the field of security. Particularly, fingerprinting and face recognition have been used for personal authentication. However, these systems suffer from certain issues, such as fingerprinting forgery, or environmental obstacles. To address forgery or spoofing identification problems, various approaches have been considered, including electrocardiogram (ECG). For ECG identification, linear discriminant analysis (LDA), support vector machine (SVM), principal component analysis (PCA), deep recurrent neural network (DRNN), and recurrent neural network (RNN) have been conventionally used. Certain studies have shown that the RNN model yields the best performance in ECG identification as compared with the other models. However, these methods require a lengthy input signal for high accuracy. Thus, these methods may not be applied to a real-time system. In this study, we propose using bidirectional long short-term memory (LSTM)-based deep recurrent neural networks (DRNN) through late-fusion to develop a real-time system for ECG-based biometrics identification and classification. We suggest a preprocessing procedure for the quick identification and noise reduction, such as a derivative filter, moving average filter, and normalization. We experimentally evaluated the proposed method using two public datasets: MIT-BIH Normal Sinus Rhythm (NSRDB) and MIT-BIH Arrhythmia (MITDB). The proposed LSTM-based DRNN model shows that in NSRDB, the overall precision was 100%, recall was 100%, accuracy was 100%, and F1-score was 1. For MITDB, the overall precision was 99.8%, recall was 99.8%, accuracy was 99.8%, and F1-score was 0.99. Our experiments demonstrate that the proposed model achieves an overall higher classification accuracy and efficiency compared with the conventional LSTM approach.

## 1. Introduction

**Figure 1:**
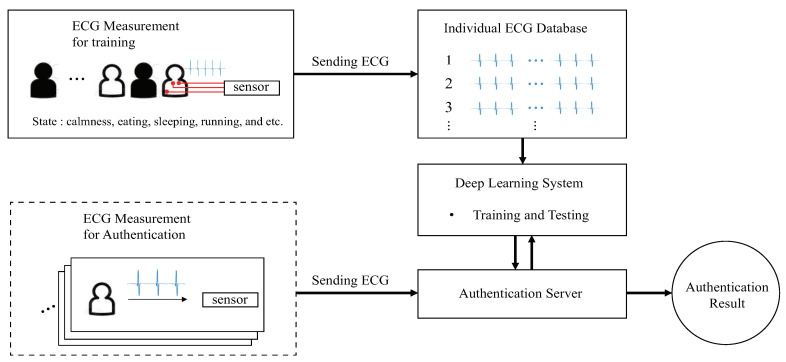
Conventional personal authentication system using ECG.

Recently, several studies involving different basic methods have been conducted in biometric systems, such as fingerprinting, face recognition, voice recognition, and electrocardiogram (ECG). However, fingerprinting and face recognition systems designed for secure personal authentication have many disadvantages, such as fingerprint forgery, or environmental obstacles, such as light, hair, or glass. Currently, voice recognition systems are commonly used for performing simple tasks, such as turning the lights off or on, making a phone call, or changing the TV channel. However, voice recognition systems are not sufficiently sophisticated to be considered as a reliable solution for an authentication system owing to the risk of spoofing with a recorded voice instead of the legitimate voice. Hence, to address forgery or spoofing identification issues, different approaches must be considered, like ECG, as presented in this paper. ECG (ECG is a test that measures the electrical activity of the heartbeat)-based biometric systems, using support vector machine (SVM), linear discriminant analysis (LDA), optimum-path forest, neural networks, and other analysis methods have been extensively studied and applied to disease diagnosis and personal authentication systems [1,2,3,4]. The aforementioned methods known as conventional ECG identification procedures are required for feature extraction that yields high accuracy in preprocessing. The recent deep learning methods do not employ feature extraction. Furthermore, to achieve a high accuracy, the deep learning methods require a lengthy input signal. The personal authentication system using ECG can be presented as shown in Figure 1.

Figure 1 shows the conventional personal authentication system using ECG with a deep learning approach. First, a personal ECG database is required that consists of all types of ECG signals that depend on the state of an individual: calmness, eating, sleeping, running, walking, etc. Then, a deep learning system is trained using the personal ECG database; consequently, an authentication server developed. The ECG signal from the dashed box in Figure 1, which is not used in deep learning, is passed to the authentication server for personal authentication. The deep learning system authenticates the user by classifying the input ECG data. This personal authentication system can be used in various self-certification services, such as automated door locks, bank vaults, and vehicles.

In this study, we propose the use of long short-term memory (LSTM)-based deep recurrent neural networks to build an ECG identification system that classifies the human ECG. The proposed method is evaluated using performance metrics by employing two public datasets from the Physionet database [5]. The major contributions of our study are as follows:

- We demonstrate the preprocessing procedures including non-feature extraction, segmentation with a fixed segmentation time period, segmentation with R-peak detection, and grouping the ECG signal of the short length. These procedures are considered for authentication time in the real-time system.
- We introduce and implement bidirectional DRNNs for ECG identification combined with the late-fusion technique. To the best of our knowledge, the proposed bidirectional DRNN model for personal authentication has not been described in the literature prior.

Further, this paper is organized as follows. Related findings in the literature are reviewed in Section 2. The proposed LSTM-based DRNN and its preprocessing for ECG are described in Section 3. Experimental results and concluding remarks are presented in Section 4 and Section 5, respectively.

## 2. Related Work

Many studies have presented different approaches designed for feature extraction and noise reduction in ECG biometrics. Particularly, Odinaka et al. explained categorizations based on features and classifiers [6]. First, the categorization requires an algorithm for feature extraction based on fiducial, non-fiducial, and hybrid features. A fiducial-based algorithm extracts temporal, amplitude, angular, or morphology features from characteristic points on the ECG data. The features extracted include the analyzed ECG information like difference in distance for each ECG wave (in the P wave, QRS complex, and T wave of ECG) [7]. Unlike the fiducial-based algorithm, a non-fiducial-based algorithm uses features, such as wavelet and autocorrelation coefficients [8,9,10]. Because such an algorithm does not use any characteristic point for developing a feature set, the detection of R peaks is required for heartbeat segmentation and alignment in most methods. A few of the remaining methods require the detection of three major components of heartbeat, such as the onset and peak of the P wave, onset and end of QRS complex, and peak and end of the T wave. A hybrid feature extraction method uses a combination of the fiducial and non-fiducial-based approaches. Moreover, the categorization requires a classifier, such as k-nearest neighbor, LDA, neural networks, generative model, SVM, and match score classifiers. The ECG can be classified using a fiducial (characteristic point), non-fiducial (similarity), and hybrid (combination of the fiducial and non-fiducial) feature extraction algorithm.

Many techniques for ECG biometric systems using various ECG databases have been proposed [11]. In [11], the authors have analyzed various studies to compare the averages of classification accuracy, identification equal error rates (EER), and authentication scenarios using normal and pathological signals ECG databases. According to their results, the weighted average rate (in an identification scenario) was 94.95% and the overall EER (in an authentication scenario) was 0.92%. Their results in [11] showed that the choice of features affects the identification accuracy rate, and the number of ECG leads used influences the performance of recognition.

In many recent studies, deep learning methods have been applied to ECG biometrics [12,13,14,15,16,17,18,19,20]. In [16], a convolutional neural network (CNN) has been used to classify patient-specific ECG heartbeats. In [17], a residual convolutional neural network (ResNet) with an attention mechanism is designed for human authentication with ECG. Unlike CNNs, a recurrent neural network (RNN) has an advantage when processing 1-D signals, such as an ECG consisting of sequential data. Generally, CNN processes 2-D data, such as an image or more 2 × 2 signal for object identification and classification, and RNN processes 1-D continuous or sequential data, such as a voice and sensor signal for identification and classification. For example, RNN has been used to classify the type of an ECG beat in [18]. However, it is difficult to train a conventional RNN using long-term sequences of data because the network develops vanishing gradients; LSTM and gated recurrent units (GRUs) have been proposed (The GRU is a modified model from LSTM) to resolve this problem [21,22]. The LSTM-based RNNs overcame the vanishing gradients and demonstrated a good performance. The LSTM-based RNNs have been widely used in applications, such as speech recognition, handwriting recognition, and ECG biometrics [23,24]. Additionally, the deep learning system can utilize the dropout technique for reducing overfitting [25]. Overfitting is observed if a deep learning model performs well while using its training dataset and it performs poorly while using its testing dataset. In [26], LSTM proved to be more suitable than GRUs for identification and classification in ECG biometrics. Thus, the LSTM-based RNNs were applied to identify and authenticate problems using ECG data [26,27,28]; deep learning techniques have shown more powerful performance compared with other non-deep learning methods.

### 2.1. Recurrent Neural Networks

**Figure 2:**
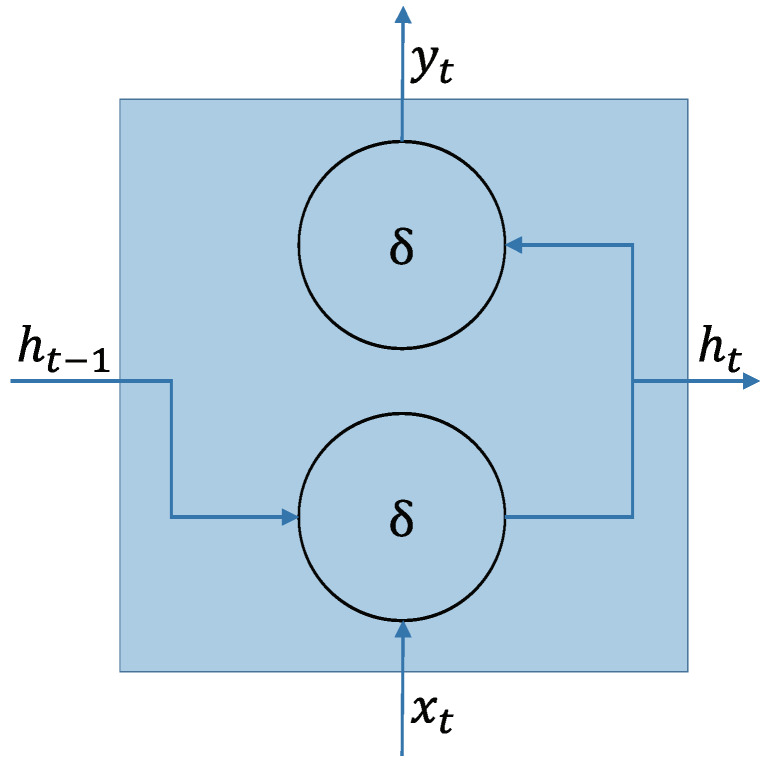
Schematic of an RNN node [29].

An RNN is a single or multiple layer neural network architecture, comprising of cyclic connections, commonly used for learning the temporal-sequential data, like string, video, and voice. This network is characterized by memorizing the instance of a previous information, which is then applied to the current input data. RNN has an advantage in handling sequential data. As shown in Figure 2, an RNN node consists of the current input $x_{t}$, output $y_{t}$, previous hidden state $h_{t-1}$, and current hidden state $h_{t}$. Thus,

(1) $$h_{t}=\delta_{hidden}\left(W_{hidden}h_{t-1}+W_{input}x_{t}+b_{hidden}\right)$$

(2) $$y_{t}=\delta_{output}\left(W_{output}h_{t}+b_{output}\right),$$

where $\delta_{hidden}$ and $\delta_{output}$ are the activation functions of the hidden layer and output layer, respectively. $W_{input}$, $W_{output}$, and $W_{hidden}$ are the weights for the input-to-hidden recurrent connection, hidden-to-output recurrent connection, and hidden-to-hidden recurrent connection, respectively. $b_{output}$ and $b_{hidden}$ are the respective bias terms for the output state and hidden state. Here, the activation function has an element-wise non-linearity feature, selected from various existing functions like the sigmoid, hyperbolic tangent, or rectified linear unit.

### 2.2. Long Short-Term Memory (LSTM)

**Figure 3:**
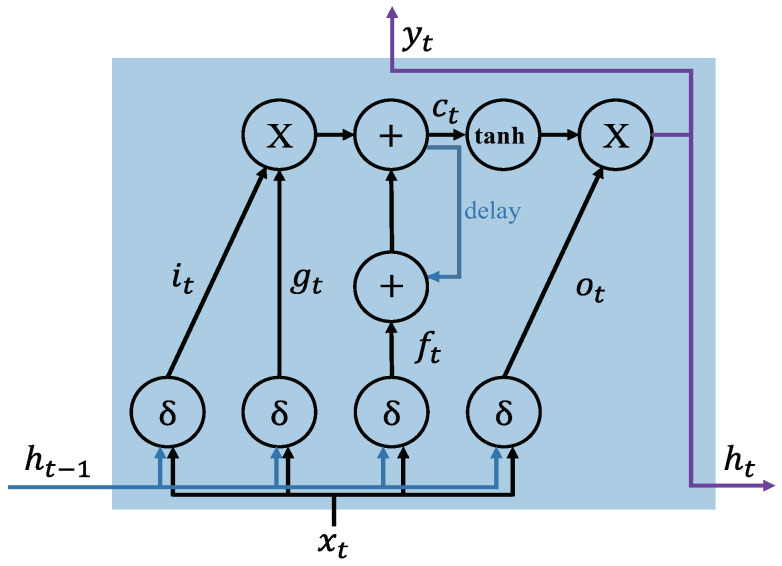
Schematic of an LSTM memory cell structure with an inner recurrence $c_{t}$ and an outer recurrence $h_{t}$. $i_{t}$, $o_{t}$, $f_{t}$, and $g_{t}$.

In conventional RNN, it can be difficult to train the long range sequential data because of vanishing or exploding gradient problems that interrupt the network’s ability to backpropagate gradients (long-term dependency problem) [30]. To solve the long-term dependency problem in the learning data, LSTM-based RNNs replace the conventional node with LSTM, which contains memory blocks with memory cells called “gates’’ in the recurrent hidden layer, as shown in Figure 3. The gates on the memory cells control the new information states updating and forgetting the previous hidden states, and determining the output. The functions of each cell component are as follows:

- Input gate ($i_{t}$) controls the input activation of new information into the memory cell.
- Output gate ($o_{t}$) controls the output flow.
- Forget gate ($f_{t}$) controls when to forget the internal state information.
- Input modulation gate ($g_{t}$) controls the main input to the memory cell.
- Internal state ($c_{t}$) controls the internal recurrence of cell.
- Hidden state ($h_{t}$) controls the information from the previous data sample within the context window:

(3) $$i_{t}=\delta \left(U_{i}x_{t}+W_{i}h_{t-1}+b_{i}\right)$$

(4) $$o_{t}=\delta \left(U_{o}x_{t}+W_{o}h_{t-1}+b_{o}\right)$$

(5) $$f_{t}=\delta \left(U_{f}x_{t}+W_{f}x_{t-1}+b_{f}\right)$$

(6) $$g_{t}=\delta \left(U_{g}x_{t}+W_{g}h_{t-1}+b_{f}\right)$$

(7) $$c_{t}=f_{t}c_{t-1}+g_{t}i_{t}$$

(8) $$h_{t}=tanh\left(c_{t}\right)o_{t},$$

where the *U* and *W* terms are weight matrices and *b* terms are bias vectors. When the LSTM-RNN trains a dataset for learning, it focuses on learning the parameters *b*, *U*, and *W* of the cell gates, as shown in (3)–(6).

### 2.3. Performance Metrics

We use four evaluation metrics measuring multi-class classification to verify the performance of the deep learning models [31].

1. Precision: it calculates the number of the true person identifications (person A, B, … G) out of the positive classified classes. The overall precision (OP) is the average of the precision of each individual class (POC: the precision of each individual class):
  (9) $$Per-POC_{c}=\frac{tp_{c}}{tp_{c}+fp_{c}}$$
  (10) $$OP=\frac{1}{C}\left(\sum_{c=1}^{C}\left(\frac{tp_{c}}{tp_{c}+fp_{c}}\right)\right),$$
  where $tp_{c}$ is the true positive rate of a person classification (*c* =1, 2,…, *c*), $fp_{c}$ is the false positive rate, and *C* is the number of classes in the dataset.
2. Recall (Sensitivity): it calculates the number of persons correctly classified out of the total samples in a class. The overall recall (OR) is the average recalls for each class (RFC: recalls for each class):
  (11) $$Per-RFC_{c}=\frac{tp_{c}}{tp_{c}+fn_{c}}$$
  (12) $$OR=\frac{1}{C}\left(\sum_{c=1}^{C}\left(\frac{tp_{c}}{tp_{c}+fn_{c}}\right)\right),$$
  where $fn_{c}$ is the false negative rate of a class c.
3. Accuracy: it calculates the proportion of correctly predicted labels (the label is the unique name of an object) as overall predictions; an overall accuracy (OA)
  (13) $$OA=\frac{TP+TN}{TP+TN+FP+FN},$$
  where, $TP=\sum_{c}^{C}tp_{c}$ is the overall true positive rate for a classifier on all classes, $TN=\sum_{c}^{C}tn_{c}$ is the overall true negative rate, $FP=\sum_{c}^{C}fp_{c}$ is the overall false positive rate, and $FN=\sum_{c}^{C}fn_{c}$ is the overall false negative rate.
4. F1-score: it is the weighted average of precision and recall.
  (14) $$F1-score=\sum_{c=1}^{C}2\left(\frac{n_{c}}{N}\right)\frac{precision_{c}\times recall_{c}}{precision_{c}+recall_{c}},$$
  where $n_{c}$ is the number of samples in a class c and $N=\sum_{c=1}^{C}n_{c}$ is the total number of individual examples in a set of *C* classes.

## 3. Proposed Deep RNN Method and Preprocessing Procedures

### 3.1. Proposed Deep RNN Method

**Figure 4:**
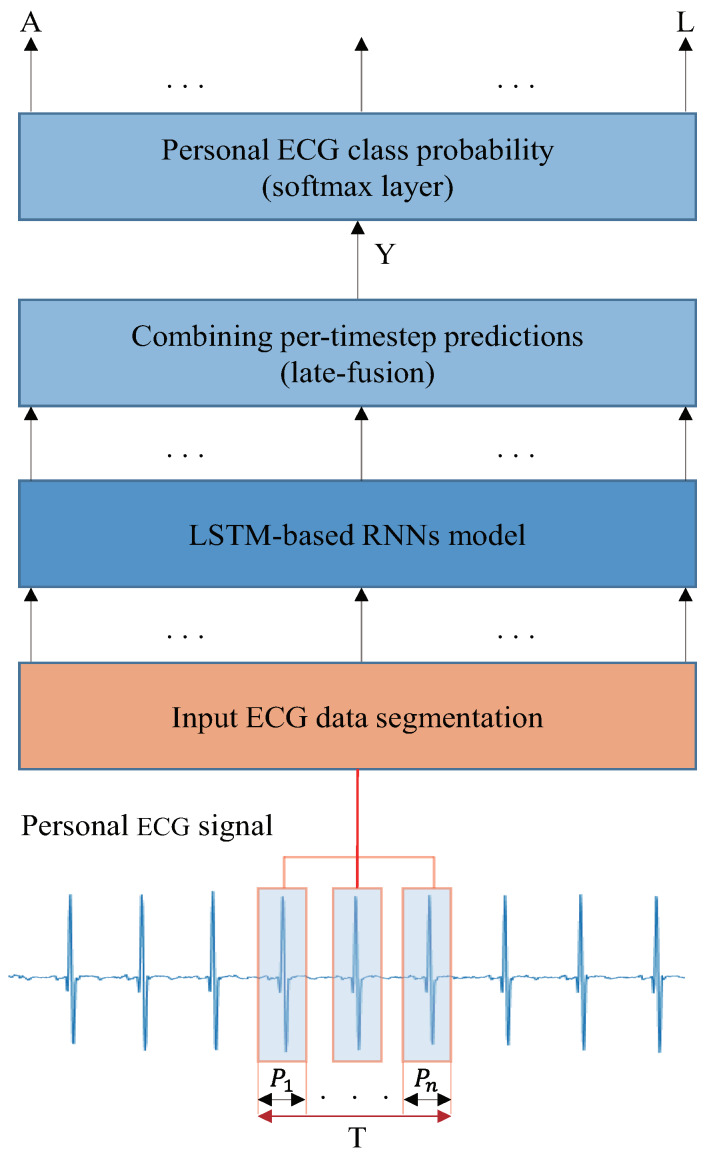
Proposed ECG identification architecture using LSTM-based RNN Model. The inputs are raw signals preprocessed from datasets, segmented into ECG components with a window size of T, and trained at the LSTM-based RNN model.

A schematic of the proposed DRNN ECG identification system is presented in Figure 4. It performs a direct mapping from personal ECG inputs to personal label classification. A specific time window is used to classify the personal labels. The input is divided into a discrete sequence of equally spaced samples $\left(x_{1},x_{2},\ldots ,x_{t}\right)$, where each data point $x_{t}$ is a vector of the personal ECG signal. The samples are passed to an LSTM-based RNN model after being segmented by the window of size T, consisting of *n* segmented ECG signal components with a period of P. In the conventional and LSTM-based RNNs, the classification accuracy is low if less than nine of ECG groups are used for training and testing [26]. In this study, we used three, six, and nine ECG groups (*n* = 3, 6, 9). In the outputs, we receive a score by denoting the personal label prediction at each time step $\left(y_{1}^{L},y_{2}^{L},\ldots ,y_{k}^{L}\right)$, where $y_{k}^{L}\in R^{c}$ is a vector of classification scores representing the given input group, *L* is for layer, and *c* is the number of person classes. The score is calculated at each time-step for the personal label at time t. The multi-prediction for the entire window T is obtained by merging all the scores into one prediction. For classification, we used a late-fusion, called “sum rule,’’ which is theoretically discussed in [32,33]. To convert the prediction scores to probabilities, we applied a softmax layer on *Y* of the prediction score.

(15) $$Y=\frac{1}{T}\sum_{t=1}^{T}y_{t}^{L}$$

**Figure 5:**
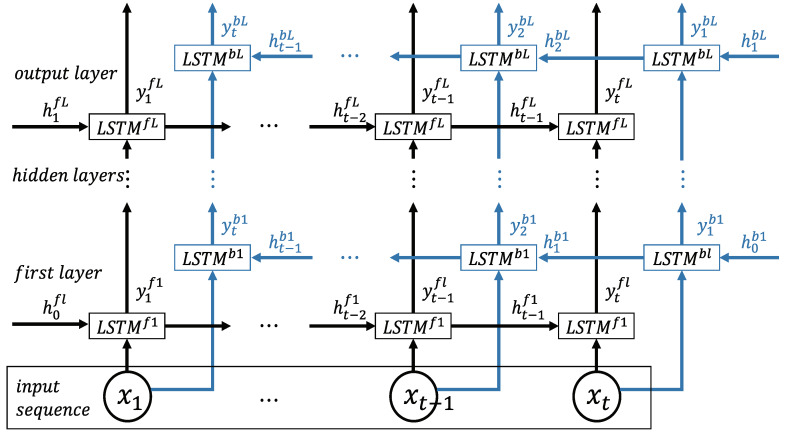
Bidirectional LSTM-based DRNN model consisting of an input layer, multiple hidden layers, and an output layer with forward $LSTM^{f1}$ and backward $LSTM^{b1}$ tracks [28].

In this study, we use bidirectional LSTM-based DRNN for further performance enhancement, as shown in Figure 5. It includes two parallel LSTM tracks: forward and backward loops for exploiting the context from the past and future of a specific time step to predict its label [28,34]. At each layer, there is a forward track ($LSTM^{fl}$) and backward track ($LSTM^{bl}$). The two tracks read the ECG input from left to right and from right to left, respectively:

(16) $$y_{t}^{fl},h_{t}^{fl},c_{t}^{fl}=LSTM^{fl}\left(c_{t-1}^{fl},h_{t-1}^{fl},x_{t};W^{fl}\right)$$

(17) $$y_{t}^{bl},h_{t}^{bl},c_{t}^{bl}=LSTM^{bl}\left(c_{t-1}^{bl},h_{t-1}^{bl},x_{t};W^{bl}\right),$$

where $y_{t}^{fl}$ and $y_{t}^{bl}$ are the output of the prediction, $h_{t}^{fl}$ and $h_{t}^{bl}$ are the output of the hidden layer, and $c_{t}^{fl}$ and $c_{t}^{bl}$ are the current output in the forward and backward layers, respectively (l=1,2,…,L). The top layer L is the output of the sequence score from the forward LSTM and backward LSTM at each time step. The combined scores $Y\in R^{c}$ represent a person label prediction score. In this case, the late-fusion is merged as follows:

(18) $$Y=\frac{1}{T}\sum_{t=1}^{T}\left(y_{t}^{fL}+y_{t}^{bL}\right).$$

To evaluate the performance of the proposed model, we perform the ECG identification experiments with six RNN structures shown in Table 1. Through the experiments, we selected Arch 6 because it results in the best identification performance.

### 3.2. Proposed Preprocessing Procedure

**Figure 6:**
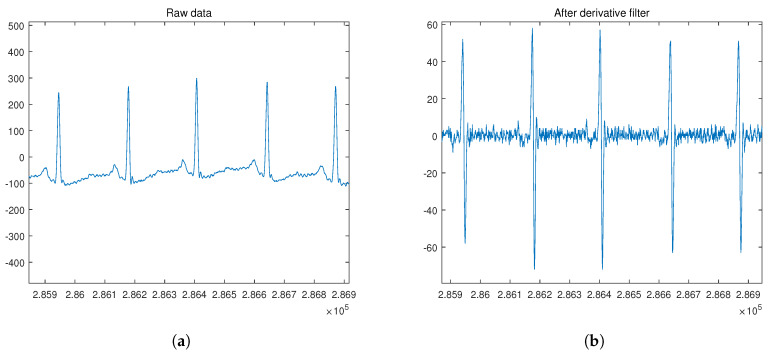

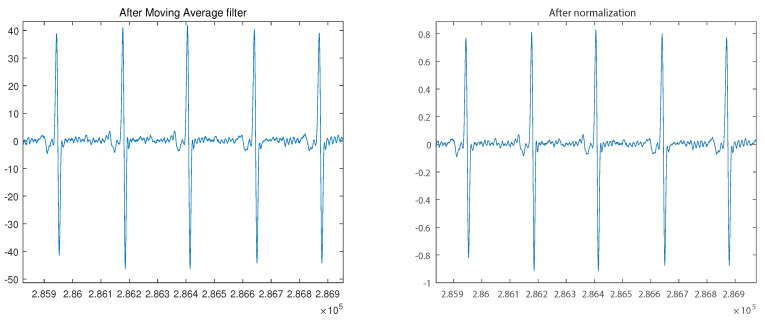
ECG signal preprocessing before the segmentation for input data: (**a**) ECG raw signal; (**b**) signal obtained after derivative filter; (**c**) signal obtained after moving average filter; (**d**) signal obtained after normalization.

The ECG database used in this study is obtained from the publicly available MIT-BIH Normal Sinus Rhythm (NSRDB) and MIT-BIH Arrhythmia datasets (MITDB), which are part of the Physionet database [35,36,37]. For the analysis, we performed the preprocessing and segmentation of each dataset. Given an ECG recording, the proposed preprocessing procedure is applied in the first step. This procedure consists of applying the derivative filter, moving average filter, and normalization for amplitude using (19) in the given order, as shown in Figure 6.

(19) $$y\left[n\right]=2\left(x\left[n\right]-x_{median}\right)/\left(x_{max}-x_{min}\right),$$

where *x*[*n*] is the *n*-th value, $x_{median}$ is the median value, $x_{max}$ is the maximum value, and $x_{min}$ is the minimum value of the input signal. The next step is to segment the ECG recordings into ECG signal components with a period of P. The conventional segmentation technique uses an R peak as a marker from the segmented individual heartbeat waveforms: P wave, QRS complex, and T wave. For the NSRDB, 288 samples were trimmed and grouped, while for the MITDB, 444 samples were trimmed and grouped.

### 3.3. Identification Procedure

In the identification procedure, each ECG dataset is divided into a training and testing set. Each training or testing sequence is of one ×*N* size, where *N* is the number of samples in the ECG signal. After one-hot sequences encoding, the weight parameters of the bidirectional LSTM are determined using the training set [38]. Then, the softmax function is used to obtain a class probability (a set of the subject probability distribution). After the RNN training, the test sequence is fed to evaluate the RNN model. A classification decision for each test sequence is obtained by selecting the class with the highest probability in all classes.

### 3.4. Dataset and Implementation

The NSRDB contains 18 two-channel recordings obtained from 18 subjects (5 males aged 26–45 and 13 females aged 20–50). Similarly, MITDB contains 48 two-channel recordings obtained from 47 subjects (25 males and 22 females). One recording for each subject was used in our proposed deep learning system. The recordings of the NSRDB were digitized using 12 bits per sample. Moreover, the recordings of the MITDB were digitized using an 11-bit resolution over a 10 mV range.

**Figure 7:**
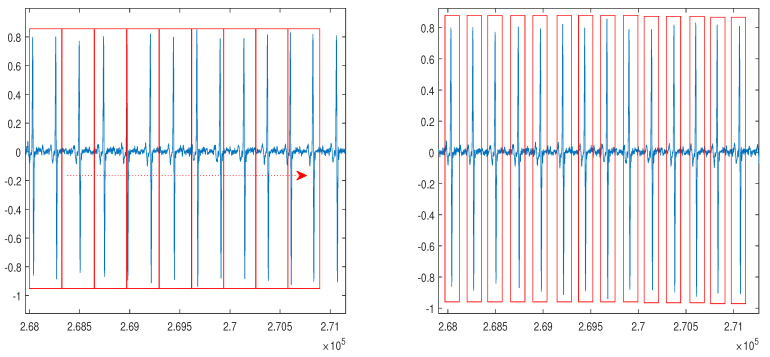
ECG signal segmentation after the normalization: (**a**) a method using a fixed segmentation time period; (**b**) a method using R-peak detection.

In our proposed method, the NSRDB and MITDB were applied in the segmentation process using the sampling frequency of the dataset. Here, the NSRDB and MITDB can be segmented using a fixed segmentation time-period or conventional R-peak detection owing to irregular ECG waveform [39]. To apply the real-time system, we considered the smallest input data size with respect to the minimum R-R interval. According to the clinical definition, the minimum R-R interval of 200 ms cannot exceed 300 bpm [40,41,42]. Thus, the selected NSRDB input size equals the time required for 288 samples (2.25 s) and the MITDB input size corresponds to the time required for 444 samples (1.23 s). Because we used the non-feature extraction method in the first experiment, the segmented data in NSRDB randomly included two to four heartbeats, and the segmented data in MITDB randomly included zero to two heartbeats as shown in Figure 7a. In the second and third experiments, we used ECG signals segmented with R-peak detection, as shown in Figure 7b.

For ECG preprocessing, particularly, to manage the generations of training and testing data, we used Matlab. The implementation, training, and testing of RNN models were performed using TensorFlow [43]. The ECG identification system uses the configuration and framework listed in Table 2. The tests were performed on our proposed model after the completion of every training epoch. We divided the processed raw data into two sets: 80% and 20% for the training and testing, respectively. The cost function used is the cross-entropy error during training, and the optimization method used is the Adam algorithm with a learning rate of 0.001 [44]. Experiment 1 was performed with a batch size of 1000, and experiments 2 and 3 were performed with a batch size of 100. The model parameters of conventional and proposed LSTM are listed in Table 3. These parameters were selected through iterative experiments using these parameters. The different conditions of the evaluation were the number of layers, number of hidden units, and input sequence length. In terms of the learning time, 4, 8, and 16 h were required for 1, 2, and 3 hidden layers, respectively.

## 4. Experimental Results and Discussion

We found various conventional classification methods being used on NSRDB and MITDB datasets. For the NSRDB dataset, the reported classification accuracy ranged from 99.4% to 100% [45,46], while for the MITDB dataset, the reported accuracy ranged from 93.1% to 100% [15,19,26,46,47,48,49,50]. The RNN-based method outperforms the aforementioned methods on both datasets. For the NSRDB and MITDB datasets, the classification experiments were performed using one recording per subject; in the NSRDB experiment, ECG signal was segmented with a fixed segmentation time period, including 2–4 training and testing beats per subject were used. Similarly, in the MITDB experiment, the unfixed group ECG including 0–2 training and testing beats per subject were used. Moreover, ECG signals segmented with R-peak detection, including three, six, and nine training and testing beats per subject were used. Because the sampling rate of the NSRDB and MITDB were different, the training and testing beats per subject were set independently for a dataset.

**Figure 8:**
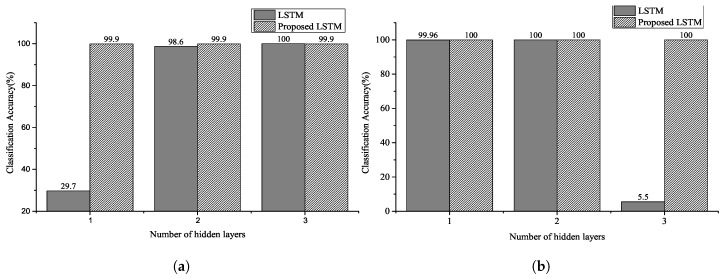
Classification accuracy for NSRDB using two selected parameters: the number of hidden unit of (**a**) is 128 and number of hidden unit of (**b**) is 250. The input sequence length is 2–4 for the number of heartbeats (Experiment 1).

**Figure 9:**
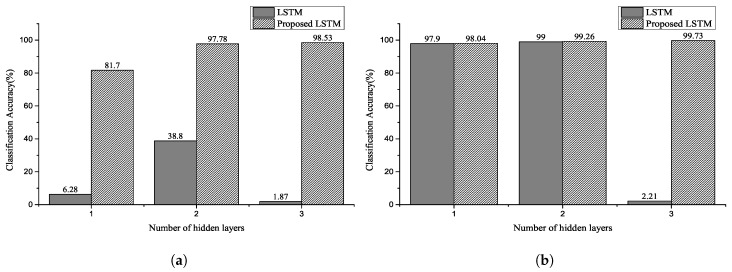
Classification accuracy for MITDB using two selected parameters: the number of hidden units of (**a**) is 128 and number of hidden units of (**b**) is 250. The input sequence length is 0–2 for the number of heartbeats (Experiment 2).

**Figure 10:**
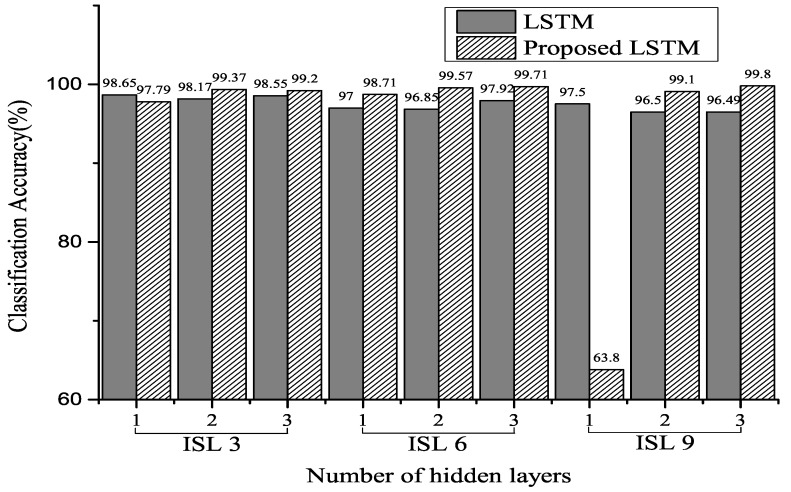
Classification accuracy for MITDB using selected parameters of 250 hidden units. The input sequence length (ISL) is 3, 6, and 9 for the number of heartbeats (Experiment 3).

Figure 8, Figure 9 and Figure 10 show the classification accuracy for the selected architectures and parameter conditions. In Figure 8a and Figure 9a, the number of hidden units of hidden layer is 128, and an ECG signal segmented with a fixed segmentation time period was used. The results of Figure 8a and Figure 9a confirm that the classification accuracy varied between 29.7–100% and 1.87–98.53%, respectively. Furthermore, in the case of Figure 8b and Figure 9b, the number of hidden units of hidden layer is 250. The results of Figure 8b and Figure 9b confirm that the classification accuracy varied between 5.5–100% and 2.21–99.73%, respectively. In Figure 10, the number of hidden units of the hidden layer is 250, and the ECG signal segmented with a fixed segmentation time period was used. Figure 10 confirms that the classification accuracy varied from 5.5–100% to 63.8–99.8%, respectively. Hence, the results presented are for different input sequence length, zero dropout, and number of hidden units. Thus, the proposed LSTM networks performed better than the conventional RNNs for the same experimental conditions. Furthermore, we can observe that a randomized decrease in the length of the input sequence—like the unfixed group ECG—improves the performance of the proposed LSTM networks and hyperparameter settings. In our experiments, the classification accuracy increased with a decrease in the number of hidden units and an increase in the number of hidden layers.

In [26], an increase in the number of the hidden layers and units increased the classification accuracy. However, in our experiments, the randomized short input sequence size—like the unfixed group ECG—resulted in an increase in the number of hidden layers and units and a decrease in the classification accuracy. Furthermore, as shown in Figure 10, the classification accuracy and number of hidden layers increased when the input sequence group size was long.

The performance results confirm that the ECG identification satisfies the LSTM and bidirectional LSTM in the NSRDB. Particularly, in our proposed model, the learning corresponded well and showed better classification results than that of the conventional LSTM model in MITDB. Table 4 and Table 5 list the performance summary for the NSRDB dataset; Table 6, Table 7 and Table 8 list the performance summary for the MITDB dataset. Table 9 shows that the proposed model outperforms other state-of-the-art methods by obtaining 99.8% classification accuracy. Although it may seem that the proposed model does not perform better than the model proposed in [26], the proposed model of [26] uses longer input sequences. However, similar to our model, when a short input sequence is used, the performance decreases to 98.2%, whilst our proposed model achieves 99.73%. Therefore, the proposed methodology yields enhanced performance, particularly with short sequences.

The primary reasons for the good performance of the proposed models for ECG classification are as follows: (1) sufficient number of deep layers enabled the model to extract personal features (2) the bidirectional model controlled the sequential and time dependencies within the personal ECG signals (3) the late-fusion technique can simplify the prediction score prior to the softmax layer step.

## 5. Conclusions

We proposed a novel LSTM-based DRNN architecture for ECG classification and performed experimental evaluation of our model on two datasets. The results confirm that the proposed model outperforms other conventional methods and demonstrates a higher efficiency. This improvement can be attributed to the ability of the model to extract more features of ECG using the deep layers of DRNN. The model can further control the temporal dependencies within the ECG signals. Furthermore, we evaluated the effect of the input sequence length and found the relationship between the hidden unit and hidden layer. The segmentation and grouping of ECG using the preprocessing procedure can effectively impact a real-time system in the classification and authentication processes. The proposed model performs better with shorter sequences compared with the state-of-the-art methods. This characteristic is useful in real-time personal ECG identification systems that require quick results. This study confirms that the proposed bidirectional LSTM-based DRNN is promising for the applications of ECG based real-time biometric identification. We lacked the scale of samples in our experiments, and the results were affected by the hardware environments. In the future, a large-scale experimentation study will be conducted with ordinary human ECG signals: calmness, eating, sleeping, running, walking, etc. Further, our proposed bidirectional LSTM-based DRNN will be extensively evaluated with other ECG signals obtained from individuals of different age groups. The future extensive research studies will aim to prove the robustness and efficiency of our proposed model.

## Figures and Tables

**Table 1:** LSTM and proposed bidirectional LSTM architectures (BiLstm).

Architectures	Layers Type
Arch 1	Lstm-softmax
Arch 2	Lstm-Lstm-softmax
Arch 3	Lstm-Lstm-Lstm-softmax
Arch 4	BiLstm-late-fusion-softmax
Arch 5	BiLstm-BiLstm-late-fusion softmax
Arch 6	BiLstm-BiLstm-BiLstm-late-fusion-softmax

**Table 2:** Server system configuration and framework.

Category	Tools
CPU	Intel i7-6700k @ 4.00 GHz
GPU	NVIDIA GeForce GTX 1070 @ 8GB
RAM	DDR4 @ 24GB
Operating System	Windows 10 Enterprise
Language	Python 3.5
Library	Google Tensorflow 1.6/CUDA Toolkit 9.0/NVIDIA cuDNN v7.0

**Table 3:** Model parameters of conventional and proposed LSTM.

Parameters	Value
Loss Function	Cross-entropy
Optimizer	Adam
Dropout	1
Learning Rate	0.001
Number of hidden units	128 and 250
Mini-batch size	1000 and 100

**Table 4:** Performance summary of the proposed bidirectional LSTM in NSRDB analysis 1 of Figure 8a.

Type of Cell/Unit	Input Sequence Length (in Number of Beats)	Number of Hidden Layer	Overall Accuracy	Overall Precision	Overall Recall	F1 Score
LSTM	2–4	1	29.7%	24.13%	29.68%	0.2662
LSTM	2–4	2	98.6%	98.73%	98.67%	0.9870
LSTM	2–4	3	100%	100%	100%	1.0000
Proposed LSTM	2–4	1	99.93%	99.92%	99.96%	0.9994
Proposed LSTM	2–4	2	99.93%	99.92%	99.96%	0.9994
Proposed LSTM	2–4	3	99.93%	99.94%	99.93%	0.9993

**Table 5:** Performance summary of the proposed bidirectional LSTM in NSRDB analysis 2 of Figure 8b.

Type of Cell/Unit	Input Sequence Length (in Number of Beats)	Number of Hidden Layer	Overall Accuracy	Overall Precision	Overall Recall	F1 Score
LSTM	2–4	1	99.96%	99.96%	99.96%	0.9996
LSTM	2–4	2	100%	100%	100%	1.0000
LSTM	2–4	3	5.5%	0.31%	0.58%	0.0058
Proposed LSTM	2–4	1	100%	100%	100%	1.0000
Proposed LSTM	2–4	2	100%	100%	100%	1.0000
Proposed LSTM	2–4	3	100%	100%	100%	1.0000

**Table 6:** Performance summary of the proposed bidirectional LSTM in MITDB analysis 1 of Figure 9a.

Type of Cell/Unit	Input Sequence Length (in Number of Beats)	Number of Hidden Layer	Overall Accuracy	Overall Precision	Overall Recall	F1 Score
LSTM	0–2	1	6.28%	7.4%	6.21%	0.0676
LSTM	0–2	2	38.80%	35.66%	38.83%	0.3717
LSTM	0–2	3	1.87%	0.06%	0.18%	0.0013
Proposed LSTM	0–2	1	81.70%	82.83%	81.68%	0.9780
Proposed LSTM	0–2	2	97.78%	97.77%	97.77%	0.9780
Proposed LSTM	0–2	3	98.53%	98.53%	98.53%	0.9855

**Table 7:** Performance summary of the proposed bidirectional LSTM in MITDB analysis 2 of Figure 9b.

Type of Cell/Unit	Input Sequence Length (in Number of Beats)	Number of Hidden Layer	Overall Accuracy	Overall Precision	Overall Recall	F1 Score
LSTM	0–2	1	99.70%	97.92%	97.90%	0.9791
LSTM	0–2	2	99.00%	99.01%	99.00%	0.9900
LSTM	0–2	3	2.21%	0.04%	2.13%	0.0008
Proposed LSTM	0–2	1	98.04%	98.07%	98.04%	0.9806
Proposed LSTM	0–2	2	99.26%	99.28%	99.26%	0.9927
Proposed LSTM	0–2	3	99.73%	99.73%	99.73%	0.9973

**Table 8:** Performance summary of the proposed bidirectional LSTM in MITDB analysis 3 of Figure 10.

Type of Cell/Unit	Input Sequence Length (in Number of Beats)	Number of Hidden Layer	Overall Accuracy	Overall Precision	Overall Recall	F1 Score
LSTM	3	1	98.65%	98.76%	98.85%	0.9981
LSTM	3	2	98.17%	98.42%	98.56%	0.9849
LSTM	3	3	98.55%	98.66%	98.86%	0.9876
LSTM	6	1	97.00%	97.37%	97.49%	0.9743
LSTM	6	2	96.85%	97.21%	97.61%	0.9741
LSTM	6	3	97.92%	98.16%	98.44%	0.9830
LSTM	9	1	97.50%	97.70%	98.07%	0.9788
LSTM	9	2	96.50%	96.69%	97.11%	0.9690
LSTM	9	3	96.49%	96.80%	97.22%	0.9701
Proposed LSTM	3	1	97.79%	98.10%	98.22%	0.9816
Proposed LSTM	3	2	99.37%	99.47%	99.52%	0.9949
Proposed LSTM	3	3	99.20%	99.30%	99.42%	0.9936
Proposed LSTM	6	1	98.71%	98.95%	99.06%	0.9901
Proposed LSTM	6	2	99.57%	99.68%	99.59%	0.9963
Proposed LSTM	6	3	99.71%	99.78%	99.72%	0.9975
Proposed LSTM	9	1	63.80%	67.31%	63.49%	0.6534
Proposed LSTM	9	2	99.10%	99.12%	99.31%	0.9921
Proposed LSTM	9	3	99.80%	99.82%	99.83%	0.9982

**Table 9:** Performance comparison with state-of-the-art models.

Methods	Dataset	Input Sequence Length (Number of Beats)	Overall Accuracy (%)
Proposed model	MITDB	3	99.73
		9	99.80
H. M. Lynn et al. [15]	MITDB	3	97.60
		9	98.40
R. Salloum et al. [26]	MITDB	3	98.20
		9	100
Q. Zhang et al. [19]	MITDB	1	91.10
X. Zhang [49]	MITDB	8	97.80
		12	98.9
Ö. Yildirim [50]	MITDB	5	99.39
